# Students’ Strategic Usage of Formative Quizzes in an Undergraduate Course in Abstract Algebra

**DOI:** 10.1007/s40753-022-00194-9

**Published:** 2022-11-09

**Authors:** Frank Feudel, Alexander Unger

**Affiliations:** Institute of Mathematics, Humboldt-Universität zu Berlin, Berlin, Germany

**Keywords:** Quizzes, Learning strategies, Students’ practices, Assessment

## Abstract

Besides homework assignments, optional quizzes are a commonly used means for formative assessment in tertiary mathematics education. Instructors, for example, implement these to help students detect gaps in their understanding, or to foster a continuous and active engagement with the content. The extent to which these goals are reached, however, strongly depends on how students actually use such quizzes, which is currently an underexplored topic. We investigated this issue in an undergraduate abstract algebra course with a study using a mixed-methods design. Unlike previous research suggesting that students use optional quizzes in tertiary mathematics courses mainly for rehearsal or for a final self-check of their own performance, our study indicates that students also use such quizzes in many other ways, for example for planning their further learning, or for deepening their understanding of the content of the course. Furthermore, our study shows differences regarding students’ quiz usage during the semester and when preparing for the final exam. Finally, the data propose factors that influence the way students use optional quizzes, for example time constraints due to other obligations during the semester, the perceived difficulty of the questions, or the opportunity to discuss these with peers. This leads to several suggestions regarding the implementation of optional quizzes into tertiary mathematics courses.

## Introduction

Quizzes are a widespread means for formative assessment in tertiary mathematics education. By “quiz”, we mean a set of questions to be answered by students, who then have to submit their set of answers, and receive feedback afterwards. The questions may ask students to either select an answer from options provided, or to give a short answer such as a number, word, phrase, or sentence. Instructors implement such quizzes for several reasons. They might, for example, help students become aware of their skills and the understanding acquired already during the semester, or they may foster an active engagement with the content (Gaspar Martins, 2017; Lim et al., 2012). Electronic quizzes can have additional advantages: they can provide students with individual feedback instantly, require fewer resources than the manual grading of homework assignments, and allow for repeated attempts (Hannah et al., 2014; Rønning, 2017). These advantages are of particular importance nowadays because the number of students entering university is increasing, so that providing individual feedback becomes a challenge for staff, especially in first-year courses (Gill & Greenhow, 2008). Furthermore, since electronic quizzes do not require any personal contact, they are a particularly fertile tool for distance learning courses (Figueroa-Cañas & Sancho-Vinuesa, 2021; Lowe, 2015), and their value for students’ assessment has grown even more during the Covid-19 pandemic.

There is some indication in the literature that a regular provision of quizzes in tertiary mathematics courses can influence students’ academic achievement positively (Angus & Watson, 2009; Chirwa, 2008; Figueroa-Cañas & Sancho-Vinuesa, 2021; Förster et al., 2018; Gaspar Martins, 2017; Hannah et al., 2014; Lim et al., 2012). However, Hannah et al. (2014) also found that just spending more time on quizzes or taking them more often does not necessarily lead to better grades. Hence, the effect of quizzes on students’ learning highly depends on how students actually use them, which is currently an underexplored topic (Walker et al., 2008). The study we present here focuses on precisely this issue, whereby we address the following question:

*How do students in a tertiary mathematics course use optional formative quizzes for their learning?*

We examined this matter in a proof-oriented undergraduate abstract algebra course, which had prospective mathematics teachers for upper secondary level as participants. In this course, optional quizzes were implemented throughout the semester. We collected quantitative data about the extent of students’ quiz usage in this course, as well as qualitative data from a survey asking *how* they used the quizzes strategically during the semester and during their preparation for the final exam. We analyzed this data with the learning strategy taxonomy by Pintrich et al., (1993). This analysis showed that our participants used the quizzes in many ways during their learning process, and not only for rehearsal, perfecting certain skills, or for checking their level of mastery of the content of the course, as has been suggested by the data of previous studies like Lowe (2015) or Hannah et al. (2014).

## Literature Review on the Usage of Quizzes in Tertiary Mathematics Education

### Instructor’s Usage of Quizzes and Their Intentions

Instructors have been using quizzes as a means for supporting students’ learning at tertiary level since the middle of the last century (Bangert-Drowns et al., 1991). Several reasons for this were proposed in the education literature already at that time (Stanlee & Popham, 1960): Quizzes might help students detect gaps in knowledge or understanding already during the semester, highlight important content of the course, motivate students extrinsically to engage with the content *continuously*, or foster an *active engagement* with the content.

The potentials of quizzes just mentioned are also common reasons why instructors of tertiary mathematics courses implement quizzes (Broughton et al., 2013; Chirwa, 2008; Gaspar Martins, 2017; Hannah et al., 2014; Lim et al., 2012). Two further important reasons have arisen with the development of digital tools for computer-aided assessment (CAA). These tools offer the possibility of providing students with *instant* feedback, which allows them to address problems detected immediately (Charman, 1999; Hannah et al., 2014; Rønning, 2017; Roth et al., 2008). Furthermore, grading no longer needs to be done by a person, which saves resources that could be used for other means of support (Chirwa, 2008; Hannah et al., 2014; Rønning, 2017). This could explain why the use of quizzes is particularly often described in articles focusing on large mathematics courses with students from several disciplines (Gaspar Martins, 2017; Hannah et al., 2014; Lim et al., 2012; Rønning, 2017).

Furthermore, quizzes are often used in courses building upon self-regulated learning, such as courses using the flipped classroom model (Cronhjort et al., 2017; Jungić et al., 2015; Maciejewski, 2015). Jungić et al. (2015), for instance, implemented pre-lecture quizzes into a first-year calculus course at a university in Canada that was organized as a flipped classroom. In this course, the students were instructed to first watch video lectures before class, and then to take quizzes on the concepts covered in order to check how well they had understood these concepts. The instructor in turn used the students’ responses in the quizzes to plan the lecture. For such courses that build upon and foster self-regulated learning, electronic quizzes have the particular value that students can decide themselves when they want to take them (Charman, 1999; Hannah et al., 2014).

Finally, since electronic quizzes do not necessarily require personal contact, they are also especially useful for distance learning or asynchronous courses (Figueroa-Cañas & Sancho-Vinuesa, 2021; Lowe, 2015), which became rather common during the recent Covid-19 pandemic.

Overall, the literature suggests that instructors of tertiary mathematics courses implement quizzes to support students’ learning for many reasons. Quizzes can encourage a continuous and active engagement with a course’s content and can help students check their knowledge or understanding already during the semester. Electronic quizzes can aid achieving these goals even more easily because feedback can be provided instantly, so that students still have their solution paths in mind. Furthermore, electronic quizzes do not require grading by people, which makes them a particularly useful tool for assessment in very large and in distance-learning courses.

### Effects of the Implementation of Quizzes in Tertiary Mathematics Courses

There is some indication in the literature that the use of quizzes in mathematics courses can have a positive effect on students’ learning. When asked, students often claim that quizzes or computer-aided assessment (CAA) questions contribute to their learning. They often highlight that CAA questions allow a rapid identification of gaps due to the instant feedback provided by CAA-systems, and that they offer the possibility of doing the same or similar problems multiple times (Broughton et al., 2012; Roth et al., 2008). Furthermore, students say that quizzes or CAA-questions motivate them to engage with the content continuously during the semester (Gaspar Martins, 2017; Roth et al., 2008). Several studies also showed that quizzes can have a positive effect on students’ academic achievement (Angus & Watson, 2009; Chirwa, 2008; Figueroa-Cañas & Sancho-Vinuesa, 2021; Förster et al., 2018; Gaspar Martins, 2017; Hannah et al., 2014; Lim et al., 2012). Since the effect of quizzes on students’ achievement is not the focus of this paper, we refrain from a detailed description of these studies here. However, we want to emphasize one important finding from the study by Hannah et al. (2014), who investigated the effect of weekly quizzes on students’ achievement in a two-semester mathematics course for engineering students. They found that the time spent on quizzes and the number of quiz attempts did not correlate with the final exam grade substantially. A multiple regression analysis even showed that the number of quiz attempts had a significant negative effect on students’ performance on their final exam. This suggests that the effect of quizzes on students’ learning strongly depends on the way students actually use them, which is our research focus.

### Students’ Usage of Quizzes

How students in mathematics courses actually use quizzes is currently underexplored. Nevertheless, there are some contributions to this issue in the literature. Lowe (2015), for example, investigated students’ engagement with online quizzes (14 optional practice quizzes and five summative quizzes) implemented into an introductory mathematics course covering basic algebra, geometry, trigonometry, and statistics at a UK university. He measured the number of attempts for each student on each quiz, the point in time of the attempt, and the score achieved. He found that participation in the quizzes decreased during the semester, and that the proportion of students taking the optional practice quizzes was relatively low except for the first weeks. Hence, only a small share of the students continuously used the optional quizzes. Some of these, however, repeated the quizzes until they got 100%, indicating that they used these for rehearsal or for perfecting certain skills. In addition, the proportion taking the optional practice quizzes covering a certain topic greatly increased shortly before the submission deadline of the corresponding summative quiz. This suggests that many students taking the optional quizzes used these for a final check of their mastery of certain content. However, there were also students who took the practice quizzes several weeks before the unit was actually taught. Lowe (2015) proposed that these students used the quizzes for checking their prior knowledge, but also for deciding which parts of the unit they wished to concentrate on. He also backed up this claim with a student’s quote from the course’s online discussion forums. Overall, the data by Lowe (2015) propose that only a small portion of students actually uses optional quizzes during their learning process, and that those who do use them might be using them mainly for rehearsal or for a final check of their mastery of the content before a summative assessment. However, since Lowe only recorded numbers of quiz attempts, and the points in time of the attempts, and did not present systematic qualitative data on students’ quiz usage except for one student’s quote from the course’s online forums, the conclusions just mentioned remain rather speculative.

Rønning (2017) carried out a study in which the way students use quizzes in tertiary mathematics courses became more explicit. He investigated students’ experiences with electronic quizzes in the courses calculus I and II for engineering students at a Norwegian university. Each quiz consisted of a set of computational problems (for instance, calculating a second partial derivative) whose solutions the students had to submit electronically. The solutions were evaluated by the CAA-system Maple T.A., which provided feedback about whether the solutions were correct or not. Rønning (2017) conducted surveys and interviews to investigate students’ experiences with these quizzes. His data indicate that many students used surface strategies to solve the quiz problems, i.e., strategies for finding correct answers that do not rely on an actual engagement with the content. Students, for instance, reported that if they could not find a solution, they just tested out different solutions, because the Maple T.A. system did neither provide feedback on their errors nor on their solution process. In addition, more than 50% of the students admitted that they sometimes copied solutions, even if the system provided a certain degree of randomization by varying parameters. Hence, the study by Rønning (2017) suggests that, when working on quizzes, students may tend to use surface strategies like testing different solutions or guessing, and are less likely to use quizzes for a deep engagement with the content. However, Rønning’s findings may have been influenced by the type of problems posed, which were mainly procedural routine tasks, as well as by limitations of the Maple T.A. system.

A study that yields very detailed results on students’ strategic usage of quizzes, but not in a mathematics course, was carried out by Walker et al. (2008). They conducted semi-structured interviews on this issue with students enrolled in different science-based courses (biology, chemistry, medicine, nursing) in which electronic quizzes, which Walker et al. called e-assessments, had been implemented. The majority of the students interviewed claimed that they used the e-assessments “to gauge the knowledge retained and to identify areas where further revision is required” (Walker et al., 2008, p. 224). Hence, these students used them for diagnostic purposes for directing further revision, but not for the learning of the actual content. Furthermore, in modules in which the e-assessment questions also counted a little for the final grade (3%), students reported taking and repeating these to attain maximal credit. One student even said to have paid little attention to the actual questions. However, some participants also used the e-assessments for a deep engagement with the content. One student, for example, claimed to have thought about a question for a long time after a first failure. Another student explained that the questions actually made her consult the textbook, which she regarded as beneficial because “it was more of a research type situation” (p. 227). Some students also mentioned that they used the feedback for learning more about the topic covered, even when the answer they had chosen was correct. Overall, the study by Walker et al. suggests that students may use quizzes not just for a final check of the knowledge retained or for rehearsal, but also for planning their subsequent learning and for deepening their understanding of a course’s content. Furthermore, the students in this study also expressed that how they engaged with the quizzes depended on the question format.

The studies mentioned previously give some indications of how students in mathematics courses might use quizzes during their learning process. However, only the study by Rønning (2017) provides explicit findings on students’ strategic usage of quizzes *in tertiary mathematics courses*, and his findings may have depended much on the quiz questions posed (mainly routine tasks). Our research therefore aims at expanding these findings by investigating *how* students use optional quizzes for their learning in an abstract algebra course focusing on mathematical concepts. Furthermore, previous research has not taken into account that students’ usage of quizzes can be different in different phases of the semester. We therefore also compared students’ usage of quizzes during the semester with how they used quizzes during preparations for the final exam.

## Theoretical Framework of the Study

Since we focused in our research on students’ usage of formative optional quizzes, we supposed that the students deliberately chose to take the quizzes or not. We therefore assumed some kind of strategic behavior, and used the notion of *(learning) strategy* as the theoretical basis for our research.

There is no unique definition of strategy in the education literature. One definition that several authors investigating students’ learning strategies have based their research on (e.g., Göller 2020; Pintrich et al., 1993; Wild & Schiefele, 1994) was formulated by Weinstein & Mayer (1986):

*Learning strategies can be defined as behaviors and thoughts that a learner engages in during learning and that are intended to influence the learner’s encoding process. Thus, the goal of any particular learning strategy may be to affect the learner’s motivational or affective state, or the way in which the learner selects, acquires, organizes, or integrates new knowledge.* (p. 315)

Weinstein & Mayer (1986) based this definition on cognitive approaches to learning. In these approaches, learning is viewed as information processing carried out by the learner actively, i.e., the learner first selects important information, and then connects it to existing knowledge by reorganizing her/his cognitive structure (see Yilmaz (2011) or Hasselhorn & Gold (2017) for more details).

However, this definition of strategy is not the only one in the literature. Indeed, there have been several proposals and discussions about how to define strategy (Bjorklund & Harnishfeger, 1990; Paris et al., 1983; Pressley et al., 1985). Paris et al. (1983), for example, who tried to characterize strategic behavior in the context of reading, argued that strategies are *deliberate actions* that are carried out *with certain intentions* and that are *actively selected between different alternatives*. A further characteristic of strategies mentioned in the literature discussing the notion of strategy is that strategies involve plans (Schmeck, 1988), and some scholars doing research on strategy use emphasized that their execution is controlled and not automized (Bjorklund & Harnishfeger, 1990).

Other scholars argued against some of these characteristics. Pressley et al, (1985), for example, who analyzed literature on children’s reading strategies, argued that a student applying a certain reading technique cannot be considered as more strategic than an expert applying the same technique automatically. They therefore emphasized that strategies are only *potentially conscious* and *potentially controllable*. The same may of course apply to techniques in mathematics, for instance when mathematics experts create examples for making sense of a new concept introduced by a formal definition (Shepherd & van de Sande, 2014). Similarly, the view that strategies need to involve planning is questionable. McDaniel & Kearney (1984), for example, have shown that students who were asked to memorize word lists applied certain memorization strategies spontaneously, for instance semantic processing of the words to be memorized. Such a spontaneous usage of strategies might also be imaginable in mathematics, for example when students are confronted with an unfamiliar task and try to apply strategies that have worked for previous tasks.

On the basis of reviewing relevant literature on learning strategies (including some of the literature just mentioned), Hasselhorn (1996) finally concluded that there is no consensus about the common characteristics of learning strategies except for their goal-directedness. He therefore formulated the following definition, which can be found in his educational psychology textbook (Hasselhorn & Gold, 2017) (translated by the authors):

*A learning strategy is composed of processes or activities that are directed towards a certain learning or retention goal, and that exceed the processes that are mandatory during the handling of a learning demand. Learning strategies have at least one additional characteristic by being either intentional, deliberate, spontaneous, selective, controlled, and/or effortful.* (p. 89)

Since we investigated students’ usage of *optional* quizzes, we assumed that at least the selectivity is fulfilled, and therefore considered students’ usage of the quizzes as strategic in this sense.

The literature provides several taxonomies for analyzing learning strategies (Hasselhorn & Gold, 2017; Mandl & Friedrich, 2006; Pintrich et al., 1993; Wade et al., 1990; Weinstein & Mayer, 1986; Wild & Schiefele, 1994). Weinstein and Mayer (1986), for example, proposed the following classification: rehearsal strategies, elaboration strategies, organizational strategies, comprehension monitoring strategies, and affective strategies. They supported this classification theoretically by explaining how these strategy types can help to master different demands for processing the information that ought to be integrated into a learner’s cognitive structure.

Pintrich et al. developed a similar taxonomy that they also validated empirically (Pintrich et al., 1993). They divided learning strategies into three basic types: cognitive strategies, metacognitive strategies, and resource management strategies. The cognitive strategies cover rehearsal strategies like repeating, elaboration strategies like paraphrasing, organizational strategies like outlining, and critical thinking. The metacognitive strategies cover the planning and setting of goals, monitoring comprehension, and regulation, i.e., adjustments if the goals set are not reached. The resource management strategies cover time management, management of the study environment, effort regulation, help-seeking, and learning with peers. Pintrich et al. used this taxonomy for developing a questionnaire for the investigation of students’ learning strategies (the MSLQ, see Pintrich (1991)), with which they checked that their taxonomy was also empirically reasonable. On the basis of this taxonomy, Wild & Schiefele (1994) developed a similar questionnaire with small adaptations to the items and some further subscales for Germany, where our study took place. They checked the adequacy of the taxonomy by administering the questionnaire to students from several study programs. A factor analysis then showed that the different categories in the taxonomy also showed up empirically as different subscales. This validated the taxonomy by Pintrich et al. (1993) also for students in Germany.

We chose this taxonomy as the theoretical basis for our research on students’ strategic usage of quizzes, because it is theoretically grounded by cognitive learning models and has been – as just described – empirically validated, also for students in Germany. Furthermore, we considered it as applicable for our context, as its categories were not too specific.

## Institutional Setting

### Description of the Course and of Its Participants

Our study took place in an undergraduate proof-oriented course “Algebra and number theory” at a large university in Germany in 2020. The topics of the course were:

1. Natural numbers (axiomatic definition, divisibility, and prime factorization).
2. Basics of group theory (groups, subgroups, homo- and isomorphisms, cosets, Lagrange’s theorem, normality, factor groups, the homomorphism theorem, the construction of groups from semigroups with cancellation laws, and the construction of the integers as a special case).
3. Basics of ring theory (rings, zero divisors, integral domains, units, subrings, ring homomorphisms, ideals, factor rings, the construction of quotient fields, and the construction of the rationales as a special case).
4. Real numbers (the construction of the real numbers using Cauchy sequences of rational numbers).

The course consisted of a lecture (two times 90 min per week) and one tutorial per week (90 min), and lasted 13 weeks. Each week, the students were obliged to complete written homework assignments, which were graded. In order to obtain admission to the final exam, which took place four weeks after the last lecture, they needed to attain 50% of the maximum points in these assignments. The written homework assignments were the only compulsory component of the course.

The course participants were prospective grammar school teachers who will later teach mathematics up to the end of secondary level. These have to take the same type of proof-oriented courses as mathematics majors in Germany. Hence, although its participants were future teachers, the course can be viewed rather as a typical proof-oriented mathematics course than as a course with a specific design for future teachers. Nevertheless, there may have been differences in the participants’ study behavior compared to that of mathematics majors, because prospective teachers in Germany also have to study a second subject, which requires further study time. The students in this course were either in their second or their third year – due to organizational issues resulting from the schedule of their second subject. Their previous experience with university mathematics was nevertheless similar, as they had all taken exactly the same courses before: Linear Algebra and Analytic Geometry I and II, Analysis I and II, and Axiomatic Geometry. 85 students actively took part in the course, i.e., they either attended the lectures or the tutorials, or submitted at least one of the written homework assignments.

Due to the Covid-19 pandemic, the course was organized as a synchronous online course in 2020. The lecturer was an experienced university mathematics lecturer, but was teaching this course for the first time. He closely followed the textbook by Kramer (Kramer & Von Pippich, 2013), who had taught the course in previous years. He decided to organize the lecture as a flipped classroom. Before the lecture, the students had to read sections from the textbook just mentioned. During the lecture, the instructor then discussed examples of the concepts covered in the respective book sections and gave additional explanations on the proofs (or proved some statements differently). The concepts introduced in the lecture were then deepened and consolidated in the weekly tutorials. These were given by the two authors who also supported the lecturer as teaching assistants. Furthermore, for fostering students’ continuous engagement with the content, and for gaining insights into students’ mastery of the course’s content during the semester in this pure online-course, formative optional quizzes on the concepts covered were implemented into this course for the first time. However, similar quizzes had already been implemented into the first-year courses Analysis I and Analysis II the year before, so most participants were already familiar with such quizzes.

### Description of the Quizzes and Their Implementation Into the Course

Two types of optional quizzes were implemented. Before describing the two in detail, we want to mention the common design principle underlying them. Both quiz types followed the idea of so-called Concept-Tests by Mazur (2017) (this term was also used in the course). These are “short multiple-choice questions on the conceptual understanding of the concept taught” (p. 10). The Concept-Tests used in our study were newly designed for the course by the staff involved, who already had experience in designing such questions. For this course, they designed the questions on the basis of literature on students’ understanding of the concepts covered, for instance Dubinsky et al. (1994) or Leron et al. (1995) for concepts of group theory, and on the basis of their teaching experience.

**Fig. 1:**
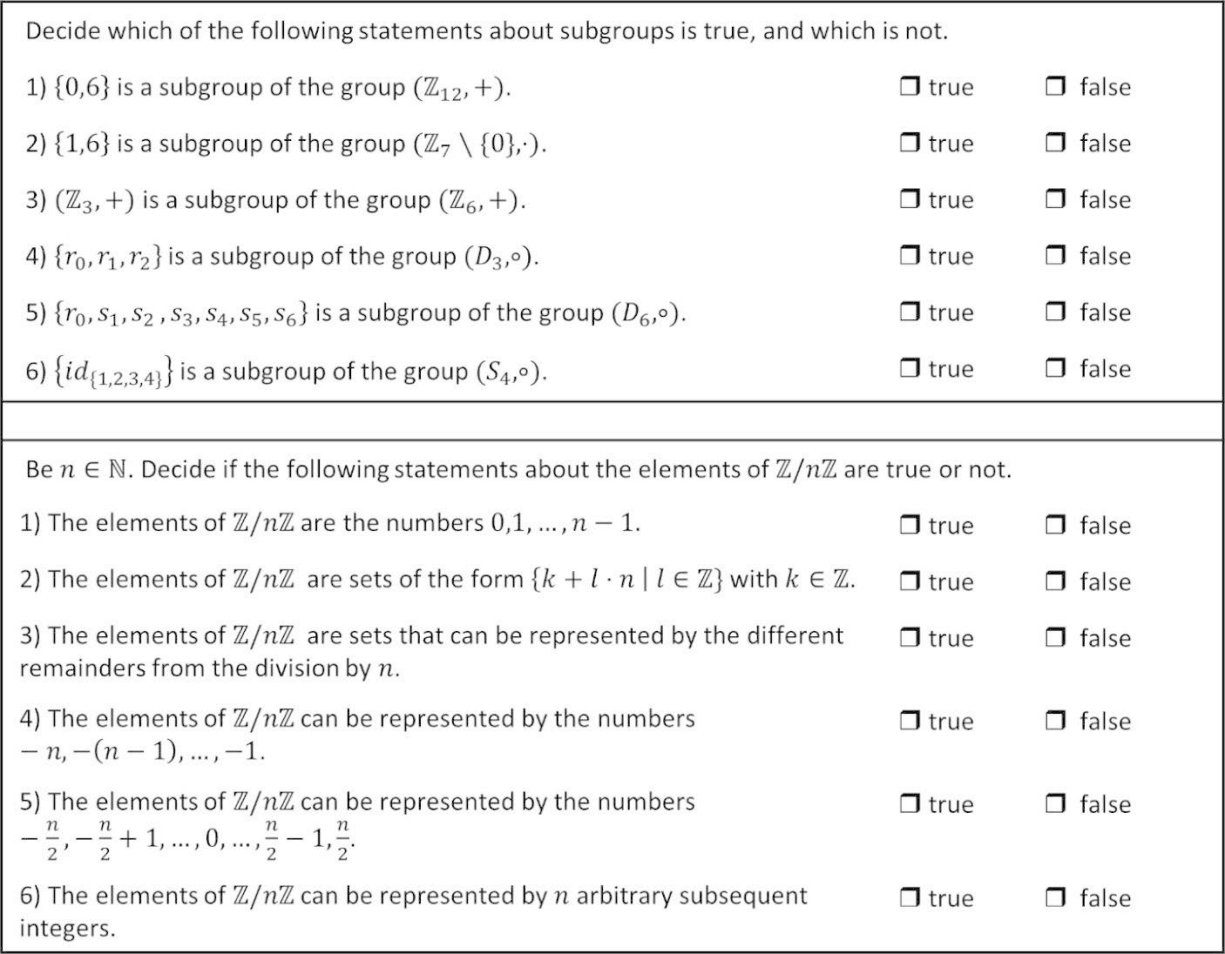
Two sample question blocks from the weekly electronic quizzes

We now describe the two quiz types in detail. One was an electronic quiz administered weekly that was implemented into Moodle – a virtual learning environment accompanying the course that contained all the course material. Each of these quizzes consisted of five theme blocks with six true/false questions (30 questions altogether). They were made available after the second of two lectures per week on Wednesdays, and could be completed until Sunday midnight. The students only had one attempt, to discourage them from guessing. Two sample question-blocks^1^ can be seen in Fig. 1. One focuses on the concept of subgroup and the properties sets need to have for being a subgroup. The other focuses on residual classes and the connection of such to their representatives. After the semester, the weekly quizzes were made available again until the final exam (this was announced only at the end of the semester). Again, the students only had one attempt.

**Fig. 2:**
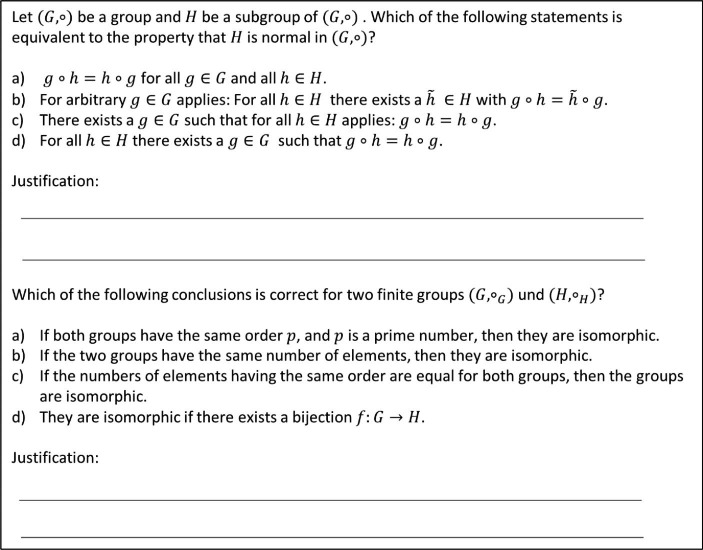
Sample questions from the “chapter finishing quizzes”

The second type of quiz were larger “chapter finishing quizzes” focusing on three of the four course chapters: natural numbers, basics of group theory, and basics of ring theory. These were made available after the chapter had been fully covered in the lecture, and could be completed within one week. They were meant to be more difficult than the weekly quizzes, because the students should have already been familiar with the topics when taking these quizzes. Therefore, *four answer options* had been designed for each question, and the students were also asked to justify their answers. The students were supposed to submit handwritten solutions for these quizzes. These were graded manually by one of the teaching assistants, who gave feedback about whether the answers and the justifications were correct and why. Two sample questions can be seen in Fig. 2: one focuses on the concept of normality and its possible confusion with commutativity, the other on the concept of group isomorphy and misconceptions about sufficient conditions for isomorphy.

Using such true/false and multiple-choice questions in optional quizzes has some advantages. Students might believe that they can solve these more quickly than open questions, which could motivate more students to take such quizzes. Furthermore, if students are not yet completely familiar with the topics, the answer options can at least provide clues for solutions, which might again motivate students to work on the questions if they do not know an answer immediately. Hence, the quiz format chosen could motivate more students to use these quizzes rather than quizzes with open questions.

However, the use of such true/false and multiple-choice questions has shortcomings. First, such questions have the disadvantage that they allow guessing. Furthermore, in multiple-choice questions, students might be attracted to the correct answer by surface properties of the answer options. In the case of the question on normality in Fig. 2, for instance, they might just remember that normality is not commutativity, and therefore already choose option b) without understanding in detail why b) is correct. This might prompt students to answer such quiz questions superficially. Finally, such questions do not ask students to formulate answers by themselves, as is usually required in exams. Hence, they might be viewed as less useful for the (final) exam than open homework assignments. Nevertheless, we considered these to be a helpful additional opportunity for engaging with the content of the course.

## Methodology of the Study

### Data Collection

We used a mixed-methods design. We collected quantitative data about the extent of students’ quiz usage from all 85 active participants. First, we recorded for all these students which of the quizzes they submitted. For the weekly electronic quizzes, we also recorded students’ processing times from first access to the questions until submission, which had been provided by Moodle.

We furthermore collected qualitative data about *how* the students used the quizzes by means of a survey. The corresponding questionnaire consisted of 14 questions altogether. Seven of these focused on students’ quiz usage during the semester, and seven on their quiz usage after the semester when preparing for the final exam. The ones focusing on students’ quiz usage during the semester were:

1. *How many of the weekly and of the chapter finishing quizzes* 2 *did you take during the semester?*
2. *If you did not use the quizzes regularly, please state a reason.*
3. *Please describe how you integrated the weekly quizzes and the chapter finishing quizzes into your study activities out of class (for example in relation to the post-class processing of the lecture, solving the written homework assignments, or preparing for the tutorials).*
4. *To what extent did you complete the quizzes intuitively, and to what extent did you think about justifications for your answers?*
5. *To what extent did you prepare or take the quizzes alone or with peers?*
6. *How did you cope with the results of the quizzes?*
7. *Describe your impression to what extent the quizzes have contributed to an understanding and to the consolidation of the content during the semester.*

Although we formulated the questions in a rather general manner, they aimed to touch on different strategies of use represented in our theoretical framework by Pintrich et al. (1993): rehearsal and elaboration (for example in the questions 4 and 7), planning and monitoring (for example in question 3), regulation (question 6), peer learning (question 5), and possibly help-seeking (question 6). Time management is touched upon in question 2, because we supposed that many students might not have taken the quizzes regularly because they did not find enough time.

Questions 8–14 focused on students’ quiz usage in the exam preparation phase. These were essentially the same as questions 1–7, except that we replaced terms like “during the semester” with terms relating to exam preparation. Asking about students’ quiz usage in two phases at the same time like this has the potential to encourage students to think about differences explicitly, although a simple repetition of responses may also be possible.

We gave out the questionnaire just described after the semester one week before the final exam. 20 students completed it. These covered a rather large spectrum of students concerning the extent of quiz usage: from students who just took some quizzes on the whole, over students who took just some quizzes during the semester but almost all quizzes when preparing for the final exam, to students who took all quizzes in both phases. Nevertheless, the small sample might have been biased. Besides this, the survey has further important limitations. First, the survey data cannot show the extent to which certain strategies were used in the whole group. Moreover, the survey participants might have also used further strategies that they did not refer to in their survey responses. Nevertheless, the data can illustrate a wide spectrum of how students in a tertiary mathematics course *might use* optional quizzes.

### Data Analysis

We analyzed the quantitative data by counting for each quiz the number of students who had submitted it, and by counting for each of the 85 participants the number of quizzes submitted during the semester and before the final exam. Furthermore, we investigated the distributions of students’ processing-times of the weekly electronic quizzes by means of box-plots.

We analyzed the qualitative data by categorizing students’ responses with content analysis – a method for analyzing communication systematically on the basis of clear coding rules (Mayring, 2015). For this, the communication is broken down into parts that are assigned to certain categories. These can be either predefined based on a theory or can emerge from the data. In our case, we investigated how students strategically used the quizzes, and therefore used the strategy types of our theoretical framework – the learning strategy taxonomy by Pintrich et al. (1993) – as categories. These were:

1. Cognitive strategies: rehearsal, elaboration, organization, and critical thinking (4 categories).
2. Metacognitive strategies: planning, monitoring, and regulation (3 categories).
3. Resource management strategies: time management, management of the study environment, effort management, peer learning, and help-seeking (5 categories).

In a first step, *the first author pre-categorized* the data according to these strategy categories by using the category descriptions from the manual of the MSLQ-questionnaire (Pintrich, 1991). Thereby, a student’s response to one question was assigned to multiple categories if different parts of it could be related to different strategies. We shall briefly illustrate this for the following sample response to the question of how students integrated the quizzes into their study activities out of class:

After I became familiar with the topic, I took the Concept-Test for testing my knowledge and for consolidation.

The first part of the response was assigned to the category “planning” because the student took the quiz on a certain occasion, the second part to “monitoring” because the student wished to check her knowledge, and the last part about consolidation was assigned to “rehearsal”.

After this first pre-categorization, we dropped those strategies from Pintrich et al. that the students did not refer to in the survey (critical thinking, management of the study environment, effort management), and refined the other strategy categories inductively according to different behavior patterns relating to these strategies that became visible in the data. This refinement covered the following steps:

1. Development of a first refinement of the categories on the basis of the data *by the first author*.
2. Categorization of *five cases by the second author* using the refinement.
3. Adaptation of the refinement on the basis of the second author’s experiences during the coding.

The resulting final category system and the corresponding coding rules can be seen in Table 1. The two authors then separately coded the whole dataset using this system. Again, a response to one question was assigned to multiple categories if different parts could be related to different activities from Table 1 – even if these belonged to the same strategy type like El1 and El2 (although the latter case never occurred). Finally, the two authors compared their results, and resolved disagreements *in a discussion*.

**Table 1:** Categories of the refined (final) category system and the corresponding coding rules

Strategy		Category name	Criteria for assigning a part of a response to the category
Cognitive strategies	Rehearsal	RH	A student claims to have used the quizzes or their solutions for revision, consolidation, memorization, or for practicing.
	Elaboration	El1	A student mentions having tried to comprehend the solutions presented or their own errors, or to connect the solutions presented to their own ones.
		El2	A student mentions having thought about justifications as to why certain answer options were correct or incorrect.
		El3	A student claims to have used the quizzes for gaining a deeper understanding of the concepts covered, or that the quizzes were useful for this.
	Organization	Org1	A student claims to have used the quizzes for gaining an overview of important topics.
		Org2	A student claims to have marked or noted down certain questions, solutions, or statements related to the quizzes.
Metacognitive strategies	Planning	Pl	A student claims to have taken the quizzes at a certain point or on a certain occasion during her/his learning.
	Monitoring	Mon	A student claims to have used the quizzes for checking her/his understanding/knowledge or for getting feedback, or that the quizzes were useful for this.
	Regulation	Reg	A student mentions having adapted her/his learning in connection with the quizzes.
Resource management strategies	Time management	TM 1	A student claims that she/he often did not have time for the quizzes.
		TM 2	A student mentions that she/he had time for the quizzes or did not have time *only on exceptional occasions*.
	Peer learning	Peer1	A student claims to have taken the quizzes solely alone.
		Peer2	A student claims to have taken the quizzes alone, but to have discussed the solutions with peers afterward.
		Peer3	A student claims to have taken the quizzes with peers.
	Help-seeking	HS1	A student mentions problems with the content of the quizzes, but did not seek any help.
		HS2	A student claims to have used media (lecture notes, the course textbook, the internet, etc.) in connection with the quizzes.
		HS3	A student claims that she/he asked other people for help in connection with the quizzes.

## Results

### The Extent of Students’ Quiz Usage

**Fig. 3:**
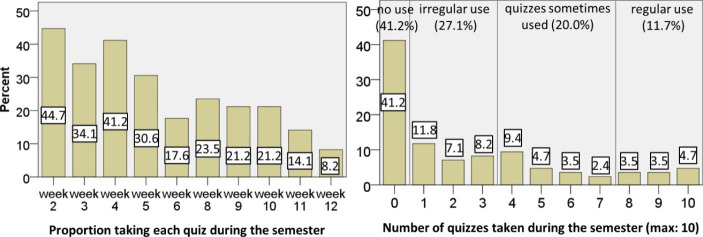
Proportion of students taking the weekly online quizzes during the semester (N = 85)

Fig. 3 shows for each of the weekly quizzes (week 7 had no quiz due to holidays) the proportion of the 85 participants who actually submitted it, as well as the number of quizzes the participants submitted in total during the semester.

This shows that a substantial proportion did not take the weekly optional quizzes, many took just some quizzes, and only a minority took the quizzes regularly during the semester. Furthermore, Fig. 3 shows that the proportion of students taking these optional quizzes decreased substantially as the semester progressed. The situation was similar for the chapter finishing quizzes. This decrease in students’ use of quizzes coincides with the results of Lowe (2015). Some of our participants also gave reasons in the survey why they had not taken the quizzes regularly during the semester. One reason was that they had not found enough time due to the obligation to submit the homework assignments (these became more difficult and probably more time-consuming from week five on) or due to duties in other courses. Others mentioned problems with the content as a reason why they had stopped taking the quizzes. But other reasons may also be possible. For example, students might have prioritized other course elements that they considered as more helpful for their learning such as the homework assignments.

**Fig. 4:**
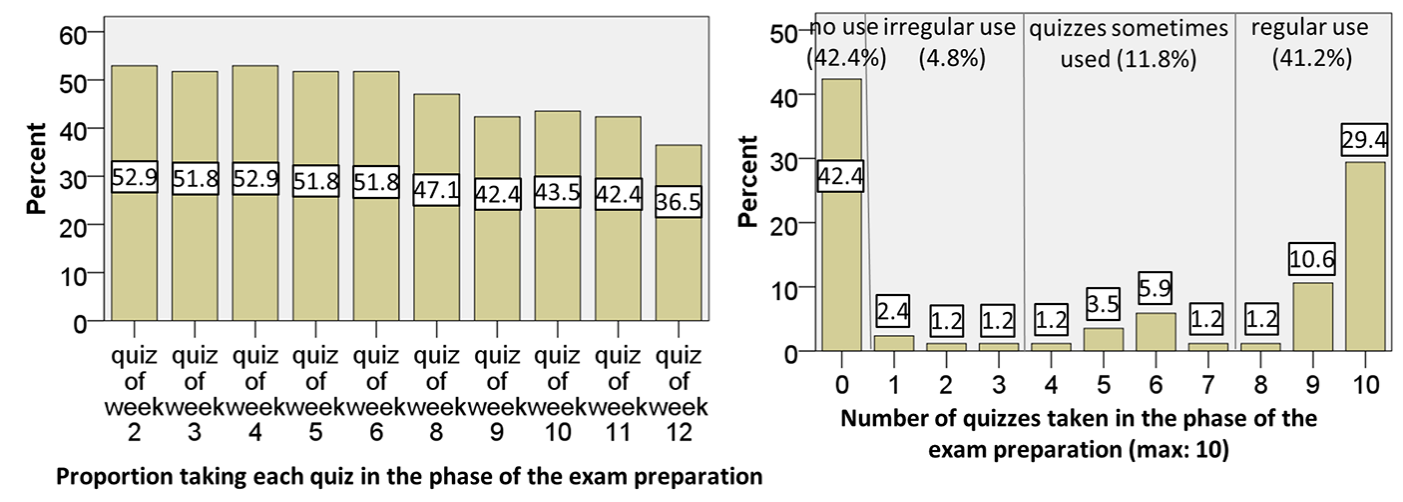
Proportion of students taking the weekly online quizzes after the semester when preparing for the exam (N = 85)

In the preparation phase for the final exam after the semester, the picture was totally different, as Fig. 4 illustrates. First, the proportion of students taking each quiz decreased much less. But more noticeably, our course participants divided basically into two groups: students not using the quizzes at all and students using all or almost all the quizzes. Nonetheless, a substantial proportion did not take any quiz. Unfortunately, our data cannot provide reasons for this behavior, which would be an important issue for future research.

**Fig. 5:**
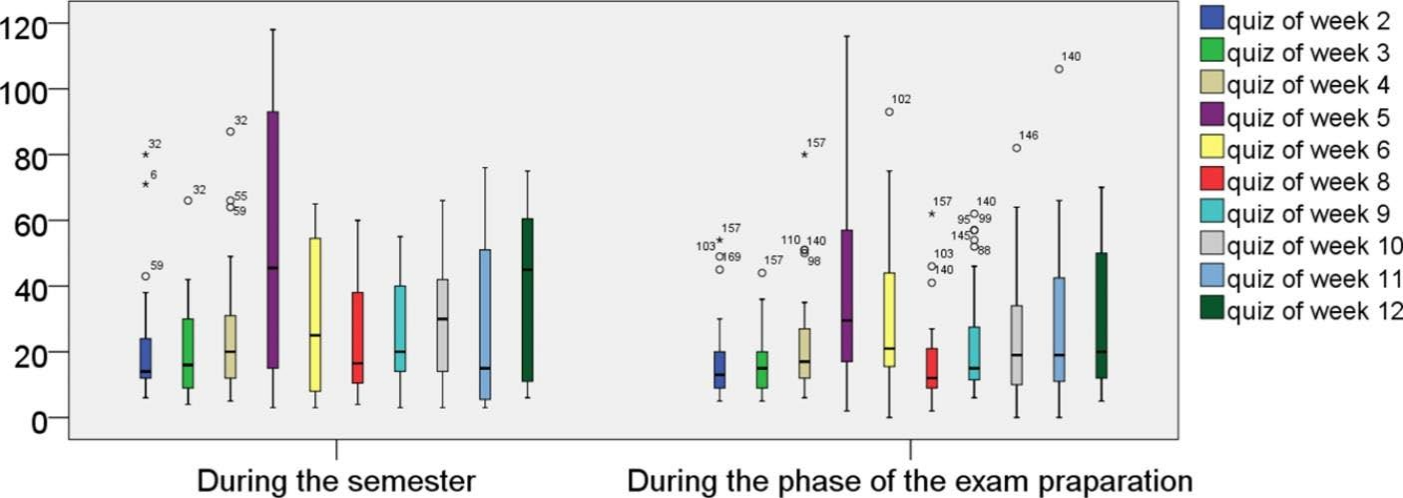
Students’ processing times (in minutes) for completion of the weekly online quizzes (N = 85)

Fig. 5 illustrates the distributions of students’ processing times of the weekly electronic quizzes from the first view of the questions until submission. These did not decrease continuously over the semester. Instead, the data suggest that the duration mostly depended on the difficulty of the topics covered in the quizzes. For the fourth and the fifth quiz, for example, the processing times were rather high. These covered cosets, normality, factor groups, and the homomorphism theorem, which rank as the most difficult topics of the course according to our experience. The same applies to the last quiz on the construction of the real numbers with Cauchy sequences. Furthermore, Fig. 5 shows that the students used less time to complete the quizzes in the exam preparation phase. One reason could be that the students were already familiar with the topics during this phase.

Overall, the quantitative data indicate that on the one hand, during the semester many students used the quizzes only occasionally, with the proportion notably decreasing over time. Students’ processing times, on the contrary, probably depended more on the difficulty of the topics covered in the quizzes. Our data also indicate that, in the preparation phase for the final exam, a substantial number of students took the quizzes on a regular basis.

### Students’ Strategic Usage of the Quizzes During the Semester

**Fig. 6:**
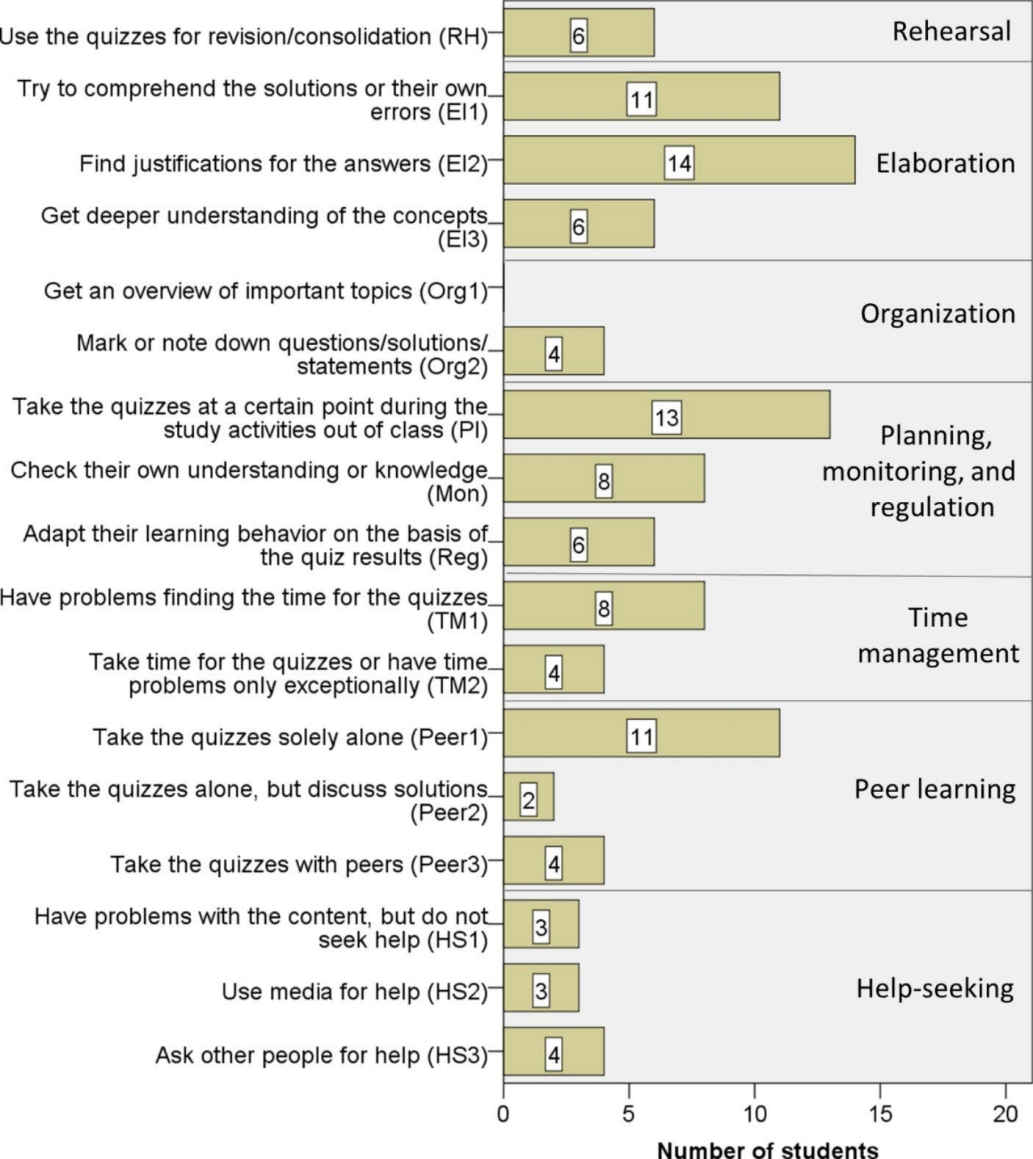
Number of students referring to the different strategies of quiz usage from Table 1 in the seven survey questions on students’ quiz usage during the semester (N = 20)

As mentioned in Sect. 5.2, we categorized the survey data on *how* the students used the quizzes according to the different learning strategies by Pintrich et al., (1993) and different behavior patterns relating to these strategies that became visible in the data (see Table 1). Fig. 6 shows the number of students referring to the different strategies and the corresponding behavior patterns at least once in the survey questions about their quiz usage during the semester. Thereby, a student was assigned to multiple categories if she/he mentioned different suitable activities. In the following, we will present examples to illustrate the different behavior patterns for each of the strategy types by Pintrich et al. (1993).

*Cognitive strategies*

#### Rehearsal

Six of the 20 students who completed the survey referred to rehearsal strategies in their responses (category “RH” in Fig. 6). They claimed that they used the quizzes for revision, consolidation, or practicing. One student, for example, responded to the question of how the students integrated the quizzes into their study activities out of class as follows:

*“At the end of the week: after the tutorials, assignments, and lectures for revision and for consolidation.”*

#### Elaboration

More students referred in their responses to elaboration strategies (see Fig. 6). First, 11 students mentioned in the question of how they coped with the results, that they tried to comprehend the correct solutions (presented after the quiz was submitted) and/or their own errors (category “El1” in Fig. 6). Regarding this activity, one important benefit of electronic quizzes (as our weekly quizzes) is that the feedback is provided instantly, as one student also mentioned explicitly:

*“It was explicitly positive that you did not have to wait long for the results, so that you still had the questions and your own thoughts in mind.”*

Second, even 14 of the 20 students who completed the survey stated that they tried to find justifications for their answers (category “El2” in Fig. 6), also in the weekly quizzes in which the provision of such was not required. Nevertheless, students were selective about whether they had tried to find justifications. One reason for not trying to find justifications was that the answer was clear:

*“I tried to find justifications for every answer, and only selected the answer option intuitively if the solution was clear to me.”*

On the other hand, some students mentioned having refrained from seeking a justification if they found a question difficult or perceived their knowledge as too low for finding one. Hence, it depended on the perceived difficulty of the questions whether our participants tried to find justifications. Another issue that influenced whether our participants sought a justification for an answer was the amount of time needed to do this. One student, for example, mentioned that he did not try to find a justification if he did not find one quickly.

Besides these specific activities (comprehending the solutions/errors, finding justifications for the answers), six of the 20 survey participants highlighted that they used the quizzes for gaining a deeper understanding of the content of the course, or that the quizzes were useful for the acquisition of such an understanding (category “El3” in Fig. 6). One student, for instance, responded to the last question about the perceived benefit of the quizzes:

*“The promotion of a deeper understanding (not just applying theorem xy on scenario z, but really understanding what happens).”*

Some students were even explicit about characteristics of the quizzes that helped them deepen their understanding. One student, for example, said that he could become aware of conceptual details:

*“I made myself aware about details: whether the objects are sets or elements, pairs of natural or integer numbers, etc.”*

Two students emphasized the usefulness of additional examples in the quiz questions, one highlighted the immediacy of the feedback that allowed a comparison with the own thoughts, and one pointed out that the questions actually demanded reflections on the content.

#### Organization

Only four students referred in their survey responses to organizational strategies (category “Org2” in Fig. 6). These mentioned to have marked/noted down certain statements considered as important, as the following responses to the question on how the students coped with their quiz results indicate:

*“For the first half of the weekly quizzes, I have marked important statements and justifications (especially also my errors) for later revision.”*

*“I noted down and collected statements that are universally valid.”*

#### Summary

Concerning cognitive strategies, the survey data indicate that our participants used the quizzes during the semester for revision and consolidation, but also for an elaboration of the content. Furthermore, the survey responses suggest that whether they used the quizzes in an elaborative manner (for instance, if they tried to find justifications for their answers) depended on the questions. Concerning organizational strategies, few participants used the quizzes to mark or note down information considered as important for their future learning.

*Metacognitive strategies*

#### Monitoring

Eight of our 20 survey participants mentioned to have used the quizzes for checking their understanding or their knowledge (category “Mon” in Fig. 6). One important reason for this was the feedback the students received, as a response to the last survey question asking how the quizzes contributed to an understanding and the consolidation of the content shows:

*“It was a further possibility to receive weekly feedback concerning the lecture content besides the homework assignments. This way, you could become aware of whether you had actually understood the material.”*

#### Planning

The responses of 13 of the 20 survey participants indicate some planning of the quiz usage, because they said to have taken the quizzes at a certain time point or on a certain occasion during their learning process (category “Pl” in Fig. 6), for instance after the post-class processing of the lecture:

*“I took them after reworking through the lecture, but before doing the homework assignments.”*

The point during their study activities out of class at which the students took the quizzes varied. The following time points were mentioned in the survey:

- before the post-class processing of the lecture,
- during the post-class processing of the lecture,
- after post-class processing the lecture, but before working on the homework assignments,
- at the very end, after the post-class processing of the lecture and after solving the homework assignments.

One student also mentioned that the point varied depending on the difficulty of the lecture (whether a revision before the quiz was necessary). This indicates that if students are offered some flexibility concerning the time point at which they can take quizzes, they may also make use of this possibility.

#### Regulation

Six of the 20 survey participants claimed to have adapted their learning in connection with the quizzes during the semester (category “Reg” in Fig. 6). One student, for example, responded to the question asking how the quizzes were integrated into the study activities out of class as follows:

*“I first tried the quizzes on Wednesdays directly after the lecture because I was curious and wanted to test myself. This worked well for the first two weeks. However, I then recognized that I could not get far without revising the lecture. In the following weeks, I at least read through the lecture content again before I tried the quiz.”*

The following regulation activities occurred in our data:

- a change of the point (during the study activities out of class) when a student took the quizzes,
- a decision to focus on other activities out of class instead of taking the quizzes,
- an adaptation of the (planned) preparation for the final exam.

#### Summary

Concerning metacognitive strategies, the survey data first indicate that our participants used the quizzes during the semester for checking their understanding or their knowledge. Second, many of them used the quizzes at a certain point during their learning process, which indicates some planning of this use. Finally, some students also adapted their learning behavior on the basis of the quiz results. However, only few adapted their learning behavior already during the semester.

*Resource management strategies*

#### Time management

Eight of the 20 survey participants mentioned that they could not take the quizzes regularly during the semester due to time problems, i.e., they did not find enough time for the quizzes (category “TM1” in Fig. 6). A sample quote from the second survey question asking for reasons why students might not have taken the quizzes regularly is:

*“No time, because of the preparation and the post-class processing of the lecture, and the need to do the homework assignments.”*

This backs up the statement already mentioned after the analysis of the Moodle data suggesting that many participants might not have taken the quizzes regularly during the semester due to time problems (see Fig. 3).

#### Peer learning

Most of our survey participants took the quizzes alone (category “Peer1” in Fig. 6). This is especially reasonable when quizzes are used as an actual self-test. However, taking quizzes with peers can also be advantageous if one wishes to use them for gaining a deeper comprehension of the content of the course, as one student emphasized:

*“I took the quizzes together with a friend, and we discussed intensively. This way, questions and reflections arose which I would not have come up with alone.”*

One important reason why many of our survey participants took the quizzes on their own might have been the Covid-19 pandemic (two students also expressed this explicitly).

#### Help-seeking

Several of our 20 survey participants made use of external help in connection with the quizzes. Three used media for help (category “HS2” in Fig. 6), for instance the lecture notes, as the following statement in a response to the question of how the students integrated the quizzes into their study activities out of class indicates:

*“I used my lecture notes while taking the chapter finishing quizzes, and I considered the results of these (‘you must practice this again’) when preparing for the exam.”*

Further media used were the course textbook or the internet. Four students asked people for help when they faced problems with the quiz content (category “HS3” in Fig. 6), for example peers or tutors.

However, the survey responses suggest that there were also students who did not seek help even though they experienced problems with the content addressed in the quizzes (category “HS1” in Fig. 6). Instead, they, for example, decided to stop taking the quizzes, as the following response to the question asking for a reason why students might not have taken the quizzes regularly, indicates:

*“I soon recognized that, during the semester, I was not fit enough in the topics for understanding the questions.”*

#### Summary

Concerning resource management strategies, our survey data suggest that many of our participants did not use the quizzes regularly during the semester because they were unable to find sufficient time. Furthermore, the responses indicate that some students might have stopped taking the quizzes when they faced problems with the content instead of seeking help. Finally, the data also suggest that those participants who had involved their peers when using the quizzes benefitted more regarding quiz usage for an elaboration of the content.

### Comparison of Students’ Quiz Usage During the Semester and in the Preparation Phase for the Final Exam After the Semester

**Fig. 7:**
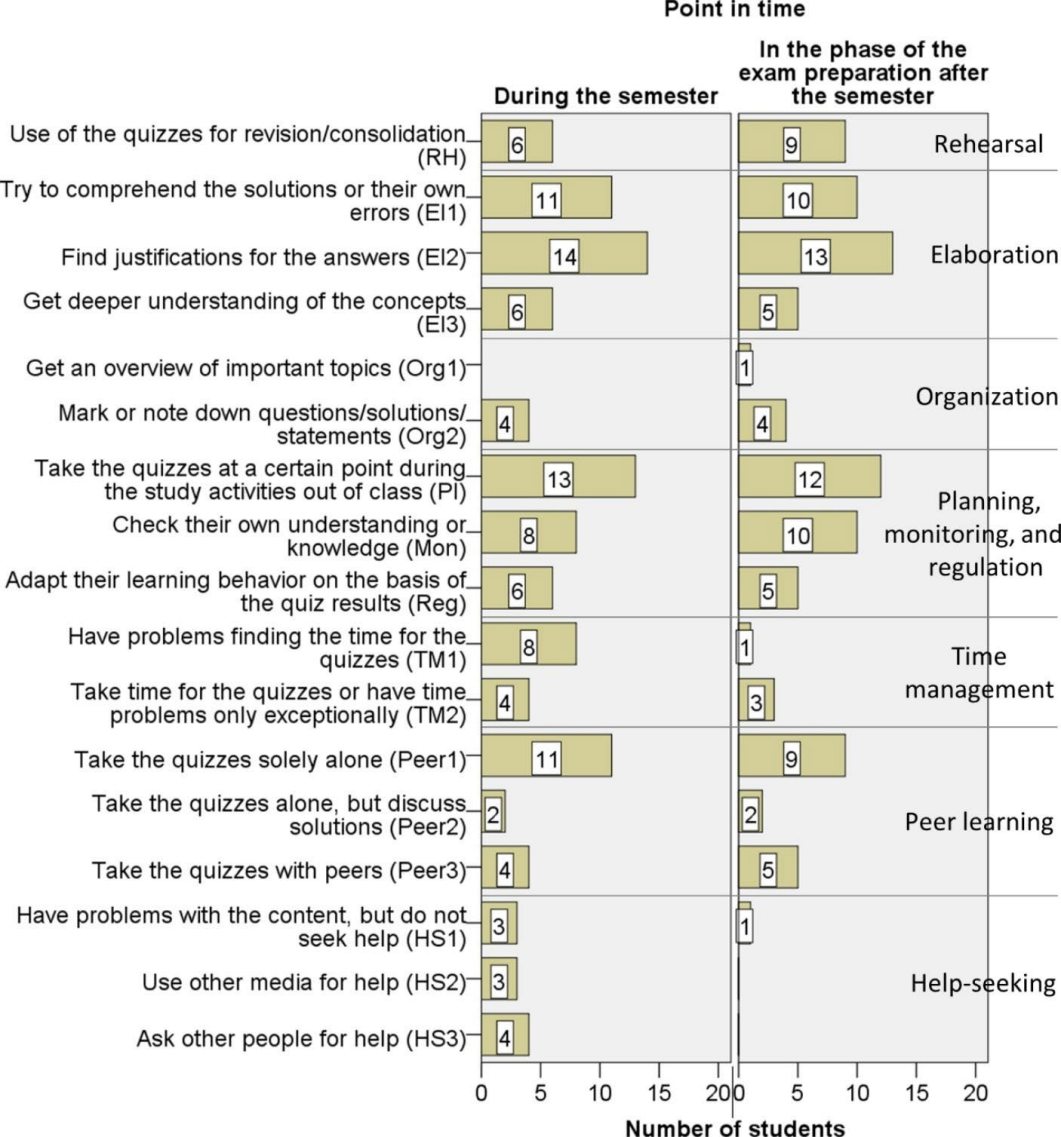
Number of students referring in the survey to the different strategies of quiz usage from Table 1 during the semester (questions 1–7) and in the preparation phase for the final exam after the semester (questions 8–14), N = 20

Fig. 7 shows the numbers of survey participants referring to the different strategies by Pintrich et al. (1993) and the corresponding patterns of use from Table 1 at least once in the survey questions during the semester and in the preparation phase for the (final) exam after the semester. We will now shortly discuss similarities and differences.

#### Comparison Regarding Cognitive Strategies

The use of the cognitive strategies was rather similar at both points in time. In the phase of the exam preparation, the survey participants referred to rehearsal strategies slightly more often (see Fig. 7). They, for instance, said to have repeated questions answered wrongly, or to have used the quizzes for memorizing definitions or theorems. Furthermore, one student now mentioned a further activity related to organizational strategies: using the quizzes as a guideline for gaining an overview of the topics to be studied (category “Org1” in Fig. 7).

#### Comparison Regarding Metacognitive Strategies

Just like during the semester, the survey participants referred to metacognitive strategies when asked how they used the quizzes in the exam preparation phase (see Fig. 7). Concerning planning, our participants again took the quizzes at different points during their learning process. In the preparation phase for the final exam, these points in time were:

- before the revision of the lecture content to find out which topics needed to be studied again,
- during the revision of the lecture content,
- after the revision and shortly before the exam as a final self-test.

Hence, our participants again made use of the flexibility concerning the time point at which they could take the online quizzes during their preparation for the final exam.

Concerning monitoring, a difference could be observed. Although the number of students referring to this issue in the survey was only slightly higher for the exam preparation phase (see category “Mon” in Fig. 7), the total number of denominations of the self-test function was much higher for this phase (18 times versus 9 times). This suggests that the use of quizzes for monitoring was more important to the students in this phase.

Concerning regulation, four survey participants now mentioned having adapted their preparations for the final exam on the basis of the quiz results, as the following response illustrates:

*“Topics that I was not fit in ought to be studied again.”*

#### Comparison Regarding Resource Management Strategies

Concerning these strategies, the data showed notable differences. First, our survey participants rarely mentioned time problems in the exam preparation phase (category “TM1” in Fig. 7). This suggests that problems of finding time for taking the quizzes mainly occurred during the semester. The second notable difference relates to help-seeking strategies (categories “HS2” and “HS3” in Fig. 7). Our participants did not mention these for the preparation phase for the final the exam. A reason might be that they now did not experience difficulties with the content. However, another reason could be that fewer opportunities for getting help were available after the semester, especially the possibility to ask tutors or peers.

#### Summary

Overall, the survey data suggest that there were many similarities concerning students’ usage of the quizzes during the semester and in the exam preparation phase, but also some differences. In particular, in the exam preparation phase, the survey participants were more likely to use the quizzes for rehearsal and for checking their mastery of the course’s content. Furthermore, in this phase, they mostly had enough time for using the quizzes and were less likely to search for help.

## Summary, Discussion, and Outlook

### Summary and Contribution to the Field

As described in Sect. 2, instructors of tertiary mathematics courses implement quizzes for a variety of reasons. Whether these intentions are fulfilled strongly depends on how students actually use quizzes, which is currently an underexplored topic. We examined this issue for optional quizzes that had been implemented into an undergraduate proof-oriented course in abstract algebra with prospective teachers for upper secondary level as participants. We first collected quantitative data about the extent of students’ quiz usage. These showed that this extent dropped noticeably during the semester, as it can be also found in the literature like Lowe (2015). During the phase of preparing for the final exam, however, this drop-off was much lower, and our course participants basically divided into those not using the quizzes at all and those taking almost all the quizzes offered.

We furthermore gathered qualitative data from a survey investigating *how* the students used the quizzes, and these findings illustrate more generally how students in tertiary mathematics courses *possibly* use optional quizzes. A summary of the cognitive and metacognitive strategies our survey participants used is shown in Table 2. This shows that they did not only use the quizzes for rehearsal, perfecting skills, or for a final check of their mastery of certain course content, as has been suggested by other studies like Hannah et al., (2014) or Lowe (2015), but also for planning their subsequent learning and for gaining an understanding of the content of the course. This dovetails with what Walker et al., (2008) found for science-based courses. But our data can also illustrate what corresponding activities in mathematics courses might look like (see right column in Table 2).

Furthermore, our data indicate that resource management strategies may affect how students use quizzes. In particular, problems of finding sufficient time or not seeking help when problems with content arose were reasons why some of our participants decided not to use the quizzes. In addition, the survey data indicate that it depends on the questions, for instance on their perceived difficulty, how students might use quizzes, which could also be hypothesized from the study by Rønning (2017) (see Sect. 2).

**Table 2:** Summary of ways in which our participants used the quizzes

Strategy		Way of using quizzes	Sample activities
Cognitive strategies	Rehearsal	Our participants used the quizzes for revision, consolidation, and memorization of the content.	They repeated questions answered wrongly or used the quizzes for memorizing definitions or theorems.
	Elaboration	Our participants used the quizzes in a way that may have helped deepen their understanding of the content.	They tried to justify their answers, used the quizzes to become aware of conceptual details and special cases, or as a source for further examples that help to get an understanding of the lecture content.
	Organization	Our participants used the quizzes for organizing the content.	They marked or wrote down certain quiz statements considered as important or noted their own errors.
Metacognitive strategies	Planning	Our participants planned the use of the quizzes during their learning process.	They took the quizzes at a certain point or on a certain occasion during their study activities out of class.
	Monitoring	Our participants used the quizzes for monitoring.	They used the quizzes for checking their understanding or their knowledge.
	Regulation	Our participants adapted their learning behavior on the basis of the quiz results.	They selected, on the basis of their quiz results, topics that they wished to revise when preparing for the final exam.

Finally, our study has given empirical evidence that students may use optional quizzes differently during the semester and during a preparation phase for the final exam. In the latter, our survey participants particularly did not mention problems to find enough time for taking the quizzes, and the self-test function of the quizzes became more important to them in this phase.

### Discussion of Implications

Our study suggests some consequences for the implementation of quizzes. First, the variety of ways our participants used the quizzes suggests that students should be given some leeway concerning the use of quizzes. For example, because the students in our sample used the quizzes at different points during their study activities out of class, students could benefit from a certain flexibility concerning when they are allowed to take quizzes, so that they can integrate the quizzes into their weekly study routine. Electronic quizzes have a particular potential for this.

Second, our data indicate that how students use quizzes depends on the nature of the quiz questions. In our study, for instance, it depended on the perceived difficulty of the questions whether the students sought justifications for their answers. The data suggest that if a question is seen as too easy or hard, students may choose to answer it intuitively, especially when they can pick from different answer options given. The same might happen if they feel the time needed to find a justification would be too great. The questions should therefore be of moderate difficulty and should not require too much time. Furthermore, our data suggest that implementing quizzes that focus on *concepts* – and not primarily on routine procedures as in Hannah et al., (2014) – might encourage students not just to use the quizzes for rehearsal or practicing, but also for gaining deeper comprehension of the course’s content. What remains open, however, is the influence of the answer format on students’ quiz usage.

Third, instructors should allocate time for the completion of quizzes within students’ regular study time out of class, because our data suggest that many students may choose not to take optional quizzes if they cannot find enough time. Allowing quizzes to be completed a second time after the semester might also help those students having time problems during the semester to still benefit from the quizzes. Nevertheless, allowing only a limited number of attempts seems reasonable, as the students in our sample rarely referred to rote-learning strategies in the survey questions focusing on the quiz usage during the semester.

Finally, if instructors pursue the goal to have students use quizzes for elaboration, our data propose that it is promising to provide them with opportunities for taking the quizzes with peers (or at least for discussing the solutions). Furthermore, it is important to offer opportunities for help, so that problems with the content of the quizzes do not induce students to stop taking these.

### Outlook for Further Research

Our research on students’ usage of optional quizzes in an abstract algebra course with prospective teachers for upper secondary level as participants illustrated empirically how students in tertiary mathematics courses *might* use optional quizzes, and gave hints about what might influence this quiz usage. This opens up several pathways for future research.

First, it is an interesting question how common strategies of use that we found are. Gathering corresponding quantitative data, and investigating students’ usage of quizzes in other courses could yield insights here. Second, it might be fruitful to explore more deeply students’ specific activities while taking the quizzes, as Dorko (2020) did for online homework. Third, our data suggest that characteristics of the questions can affect students’ quiz usage, for instance their perceived difficulty. The answer format probably also influences students’ quiz usage, for example open versus multiple-choice questions. It is important to specify such influences in the future. Finally, it would be interesting to explore further to what extent quizzes focusing on mathematical concepts (like those in our study) actually enhance students’ understanding of these. Although the pass rate of our course was, at 73%, relatively high for an abstract algebra course with prospective teachers as participants, our data cannot specify the influence of the quizzes. Hence, it would be worthwhile investigating this issue systematically, and to design questions that demonstrably improve students’ understanding of certain mathematical content. If this succeeds, quizzes can be a promising means for helping students check and enhance their understanding of mathematical content taught.

## Footnotes

1 The questions and all material from the empirical study were translated from German into English for this paper.

2 We used the term “Concept-Tests” on the questionnaire, as the quizzes were called like this in the course.
